# Characterization of the Hemagglutinin Gene of *Morbillivirus canis* in Domestic Dogs from the Mid-Western Area of Brazil

**DOI:** 10.3390/vetsci12100948

**Published:** 2025-09-30

**Authors:** Mayara Lima Kavasaki, Aneliza de Oliveira Souza, Amanda Noeli da Silva Campos, Isis Indaiara Gonçalves Granjeiro Taques, Rachel Vieira Paes de Barros, Sofia de Souza Pereira Gomes, Nathalia Assis Pereira, Tayane Bruna Soares Magalhães, Edson Viana Massoli Junior, Lucas Avelino D. Pavelegini, Luiz Donizete Campeiro Junior, Bruno Gomes de Castro, Michele Lunardi, Daniel Moura de Aguiar

**Affiliations:** 1 Marechal Rondon Faculty, Veterinary Medicine College, Vilhena 76980-000, RO, Brazil; mayarakawa@gmail.com (M.L.K.); donizete.vet@gmail.com (L.D.C.J.); 2 Laboratory of Virology and Rickettsiosis, Faculty of Veterinary Medicine, Federal University of Mato Grosso State, Cuiabá 78600-000, MT, Brazil; aneliza-oliveira@hotmail.com (A.d.O.S.); amanda.noeli@hotmail.com (A.N.d.S.C.); isis_indaiara@hotmail.com (I.I.G.G.T.); rachelvieirab@gmail.com (R.V.P.d.B.); sofiasouzapg@gmail.com (S.d.S.P.G.); nathaliaassis89@gmail.com (N.A.P.); tayane.bruna16@hotmail.com (T.B.S.M.); edson.massoli@gmail.com (E.V.M.J.); lucasavelinodandolini@gmail.com (L.A.D.P.); 3 School of Agricultural, Biological and Engineering Sciences, University Center of Várzea Grande, UNIVAG, Várzea Grande 78118-900, MT, Brazil; 4 Veterinary Medicine College, UFMT, Sinop 78550-728, MT, Brazil; bruno.castro@ufmt.br; 5 Post Graduate Program in Animal Health and Production, Department of Agrarian Sciences, University Pitagoras Unopar, Arapongas 86702-670, PR, Brazil; michelelunardi@gmail.com; 6 Laboratory of Animal Virology, Department of Veterinary Preventive Medicine, Londrina State University, Londrina 86057-970, PR, Brazil

**Keywords:** canine distemper, genetic lineages, RT-PCR, phylogenetic analysis

## Abstract

**Simple Summary:**

Canine distemper is a serious and often fatal disease that affects domestic dogs and wild animals. It can cause respiratory problems, neurological symptoms, and death. In this study, we examined brain samples from dogs that died with signs of nervous system disease in two regions of Brazil. We looked for the virus that causes canine distemper and studied the genetic material of one of its proteins, which helps the virus enter the animal’s cells. Our results showed that the virus found in these animals is related to strains already circulating in Brazil, Argentina, and Uruguay. By understanding the genetic differences between strains of this virus, we can better track its spread, monitor the risks for domestic and wild animals, and improve prevention strategies such as vaccination. This research helps protect animal health and supports public health efforts, especially in areas where the canine distemper virus is common.

**Abstract:**

Canine distemper virus (CDV) is a serious and often fatal disease caused by *Morbillivirus canis*, which affects domestic dogs and wild carnivores, with case-fatality rates reaching up to 47%. The hemagglutinin (H) protein mediates viral adsorption and shows high genetic variability, making it a valuable molecular marker. This study aimed to detect and characterize the H gene of CDV strains from 14 dogs with fatal neurological disease in the Brazilian states of Mato Grosso and Rondônia. Brain tissue was tested via RT-PCR for the nucleocapsid gene, and positive samples were amplified for the H gene. Ten complete H-gene sequences were obtained. Phylogenetic analysis revealed two distinct clusters within the South America I/Europe lineage: one related to strains from Uruguay and Argentina (with residues 530G/549Y) and another related to Brazilian strains (530S/549Y). One sequence (MT8) showed an intermediate position in the haplotype network but clustered phylogenetically with Uruguay/Argentina-related strains. Most sequences carried 530S/549Y, a pattern linked to altered SLAM receptor usage in wildlife. These findings demonstrate the co-circulation of two CDV clusters in Central–Western Brazil, their regional and international genetic connectivity, and amino acid substitutions potentially influencing host adaptation and antigenicity.

## 1. Introduction

*Morbillivirus canis*, commonly known as canine distemper virus (CDV), is a member of the order Mononegavirales within the family *Paramyxoviridae* and subfamily *Orthoparamyxovirinae* [1]. In dogs, CDV causes a multisystemic disease, with clinical signs ranging from gastrointestinal (up to 43%) and respiratory (up to 18%) involvement to neurological manifestations (~70%) and altered mental status (~53%); ophthalmic and dermatological signs are also reported (~25% for dermatological signs). Overall, case fatality rates may reach ~47% [2,3]. The virus infects multiple species within the order Carnivora, including domestic dogs (*Canis lupus familiaris*, family Canidae), as well as various wild species from the Felidae, Procyonidae, Mustelidae, Hyaenidae, Ursidae, Viverridae, Cercopithecidae, Myrmecophagidae, Elephantidae, and Phocidae families [4,5,6,7,8,9]. The disease is endemic in Brazil and, as in many countries worldwide, is primarily prevented through the use of attenuated vaccines [10]. Controlling viral diseases at the wildlife–domestic animal interface is an increasing global priority, and targeted research and surveillance are essential to anticipate spillover and guide control strategies [11,12].

The canine morbillivirus genome encodes six major proteins: nucleoprotein (N), phosphoprotein (P), matrix protein (M), fusion protein (F), large polymerase protein (L), and hemagglutinin (H). Among these proteins, M, F, H, and N are structural, whereas P and L are involved in viral replication and transcription [13]. The H protein is a type II integral membrane glycoprotein that determines viral cytopathogenicity and tropism. It is subject to strong selective pressure, particularly at amino acid residues 530 and 549, which can vary by up to 10% among CDV isolates [14]. This protein plays a critical role in host range expansion and interspecies transmission [15]. Based on genetic variation in the H gene, CDV is currently divided into seventeen phylogenetic lineages. In the Americas, lineages commonly identified in domestic and wild animals include America I to V, Rockborn-like, South America I/Europe, and South America II and III [16]. Additionally, subgenotypes have been reported within these lineages, especially among American and European strains [14].

Understanding the circulating CDV strains in Central Brazil—particularly in Mato Grosso (MT) and Rondônia (RO)—is essential to identify urban foci and mitigate spillover to wildlife, given frequent dog–wildlife interfaces; in MT, recent detections in anteaters (*Tamandua tetradactyla* and *Myrmecophaga tridactyla*) underscore this risk [4,5]. Because data on circulating strains in MT and RO are limited, we analyzed CDV from naturally infected dogs with neurological signs using N-gene RT-PCR followed by H-gene sequencing. We hypothesized that distinct South America I/Europe CDV groups co-circulate in MT and RO and harbor H-protein substitutions at residues 530/542/549 with potential effects on host range and antigenicity. We aimed to detect CDV and generate complete H-gene sequences; define their phylogenetic relationships to regional and international strains; describe key H-protein residues (530/542/549); and discuss the epidemiological relevance of these findings, including the potential for wildlife spillover.

## 2. Materials and Methods

### 2.1. Samples

Samples of central nervous system (CNS) tissue—specifically cerebellum and medulla oblongata—were collected from 14 domestic dogs naturally infected with CDV. The samples were obtained postmortem, between August 2018 and April 2019, at veterinary hospitals and clinics in the states of Mato Grosso (MT) and Rondônia (RO), Brazil. The dogs originated from the following municipalities:

- Poconé, MT (−16.2715, −56.6062): samples MT1, MT2, MT3, and MT4;
- Cuiabá, MT (−15.5997, −56.0791): MT5 and MT6;
- Sinop, MT (−11.8574, −55.5012): MT7, MT8, MT9, MT10, MT11, and MT12;
- Ji-Paraná, RO (−10.8791, −61.9442): RO1 and RO2.

### 2.2. RNA Extraction

To extract viral RNA, brain tissue samples were macerated in sterile phosphate-buffered saline (PBS), pH 7.2, and supplemented with 1% antibiotic–antimycotic solution (Sigma-Aldrich, St. Louis, MA, USA). The homogenates were centrifuged at 800× *g* for 10 min at 4 °C, and the resulting supernatants were used for RNA extraction. Viral RNA was isolated using the ReliaPrep™ RNA Cell Miniprep System (Promega, Madison, WI, USA), following the manufacturer’s instructions. An attenuated vaccine strain was included as a positive control, and ultrapure water was used as a negative control.

### 2.3. Primers

The primers used for amplification of fragments from the N and H genes were synthesized based on previously published sequences by Budaszewski et al. [14], An et al. [17], Frisk et al. [18], Harder et al. [19], and Hashimoto et al. [20]. The sequences and references for each primer set used in the N- and H-gene amplifications are detailed in Table 1.

### 2.4. One-Step RT-PCR Reaction for Canine Distemper Virus Amplification (RT-PCR)

Reverse transcription and amplification of CDV RNA were performed using the OneStep AccessQuick™ RT-PCR System (Promega, Madison, WI, USA), in a final reaction volume of 50 µL. The protocol followed the manufacturer’s instructions and consisted of an initial reverse transcription step at 45 °C for 40 min, followed by an initial denaturation at 94 °C for 3 min, and then 30 cycles of denaturation at 94 °C for 30 s, annealing at 50 °C for 30 s, extension at 72 °C for 2 min, and a final extension at 72 °C for 7 min. The CDV-1F/CDV-2R primers were used to amplify the N gene, and RH3-F/RH4-R was used to amplify the H gene.

A nested PCR was performed to enhance the amplification of the H gene using 3 µL of the first-round product. The reaction was carried out with GoTaq™ Green Master Mix (Promega), according to the manufacturer’s recommendations. Cycling conditions were the same as described above, except for the extension step, which was reduced to 1 min at 72 °C.

### 2.5. Nucleotide Sequencing, Phylogenetic Analysis, and Characterization of the H Gene

Amplified products of the N and H genes were purified using the ReliaPrep™ DNA Clean-up and Concentration System (Promega, Madison, WI, USA) and sequenced using the BigDye Terminator v3.1 Cycle Sequencing Kit (Applied Biosystems, Waltham, MA, USA) on an ABI 3500 Genetic Analyzer, according to the manufacturer’s protocol. The N-gene sequences were processed and analyzed using Geneious Prime^®^ 2024.0.7 and compared with reference sequences of CDV isolates available in GenBank.

Overlapping H-gene fragments were assembled using the ‘Contig Assembly’ tool in Geneious Prime^®^ 2024.0.7, with default parameters. The nucleotide sequences of H-gene amplicons were assembled and aligned using MAFFT v7.x, and subsequently compared with each other and the 25 international reference sequences retrieved from GenBank, including representatives of genotypes America I, II, and III; South America I/Europe; South America II and III; Europe; and vaccine strains. These sequences correspond to samples obtained from free-ranging anteaters in the study region (Mato Grosso state, MT); from dogs in the southern Brazilian states of Paraná (PR) and Rio Grande do Sul (RS); and from specimens collected in Argentina (ARG), Colombia (CO), Uruguay (UY), Italy (ITA), Denmark (DK), and the United States (US). The dataset also includes sequences from raccoons in the US and commercial vaccine strains.

A total of 35 nucleotide sequences (including the 10 sequences generated in this study) were used in the analysis, each with 1696 aligned positions. Phylogenetic reconstruction was performed using the Neighbor-Joining (NJ) method [21], with 1000 bootstrap replicates to assess clade support [22]. Evolutionary distances were computed using the Kimura 2-parameter model [23], expressed as the number of base substitutions per site. The codon positions included were first, second, third, and non-coding, and ambiguous positions were removed using the pairwise deletion option. All evolutionary analyses were conducted in MEGA v10.2.6 [24,25].

To assess potential host-specific signatures, amino acid residues at positions 519, 530, 542, and 549 of the H protein were identified and compared with those found in Brazilian Pilosa species previously infected by CDV [4,5]. The GenBank accession numbers of the reference sequences used for comparison were MG827090 (*Tamandua tetradactyla*) and MN208239 (*Myrmecophaga tridactyla*).

### 2.6. Phylogenetic and Genetic Population Network Analysis

To infer evolutionary relationships between CDV isolates and assess the phylogenetic organization of the haplotypes and the relationship between amino acid residues at critical positions (519, 530, 542, and 549), two haplotype networks were generated using the SplitTree CE v6.3.30 and PopART v1.7. The Neighbor-Net method [26,27] was implemented in SplitsTree CE v6.3.30 [28]. This reticulate network accounts for potential recombination and conflicting phylogenetic signals. The input dataset comprised 35 taxa and 35 DNA sequences, each 1696 bp in length. The P-distance method [29] was applied (default settings) to generate a 35 × 35 pairwise distance matrix. A bootstrap analysis with 100 replicates was performed in SplitsTree to assess the robustness of phylogenetic splits.

A haplotype network was generated using PopART v1.7 [30,31]. A median-joining network was constructed from the same aligned H-gene dataset, with epsilon set to 0 to prioritize parsimony. Haplotypes were defined based on single-nucleotide polymorphisms (SNPs), and the resulting network was used to visualize haplotype diversity and genetic connectivity between CDV isolates.

All networks were visualized with taxa labeled by GenBank accession number, host species, locality, and isolate ID.

## 3. Results

All samples analyzed tested positive for the *N* gene, confirming CDV infection. The sequences obtained varied in length from 208 to 281 nucleotides. Sequencing revealed 100% identity between all samples. When compared with reference sequences available in GenBank, the *N*-gene sequences from this study showed 98.2–100% similarity. The partial N-gene sequences generated in this study were deposited in GenBank under accession numbers MT119982–MT119992.

Amplification of the H gene was successful in 10 out of 14 samples (MT1, MT2, MT3, MT4, MT5, MT6, MT7, MT8, MT10, and RO2). Although both RO1 and RO2 tested positive for the N gene, only RO2 yielded a complete H-gene sequence. The H-gene nucleotide sequences ranged from 1792 to 1803 nucleotides and exhibited 99–100% similarity between themselves. The corresponding H-gene sequences were deposited in GenBank under the following accession numbers: CDV/MT1: MT119972; CDV/MT2: MT119973; CDV/MT3: MT119974; CDV/MT4: MT119975; CDV/MT5: MT119976; CDV/MT6: MT119977; CDV/MT7: MT119978; CDV/MT8: MT119979; CDV/MT10: MT119980; and CDV/RO2: MT119981.

**Figure 1:**
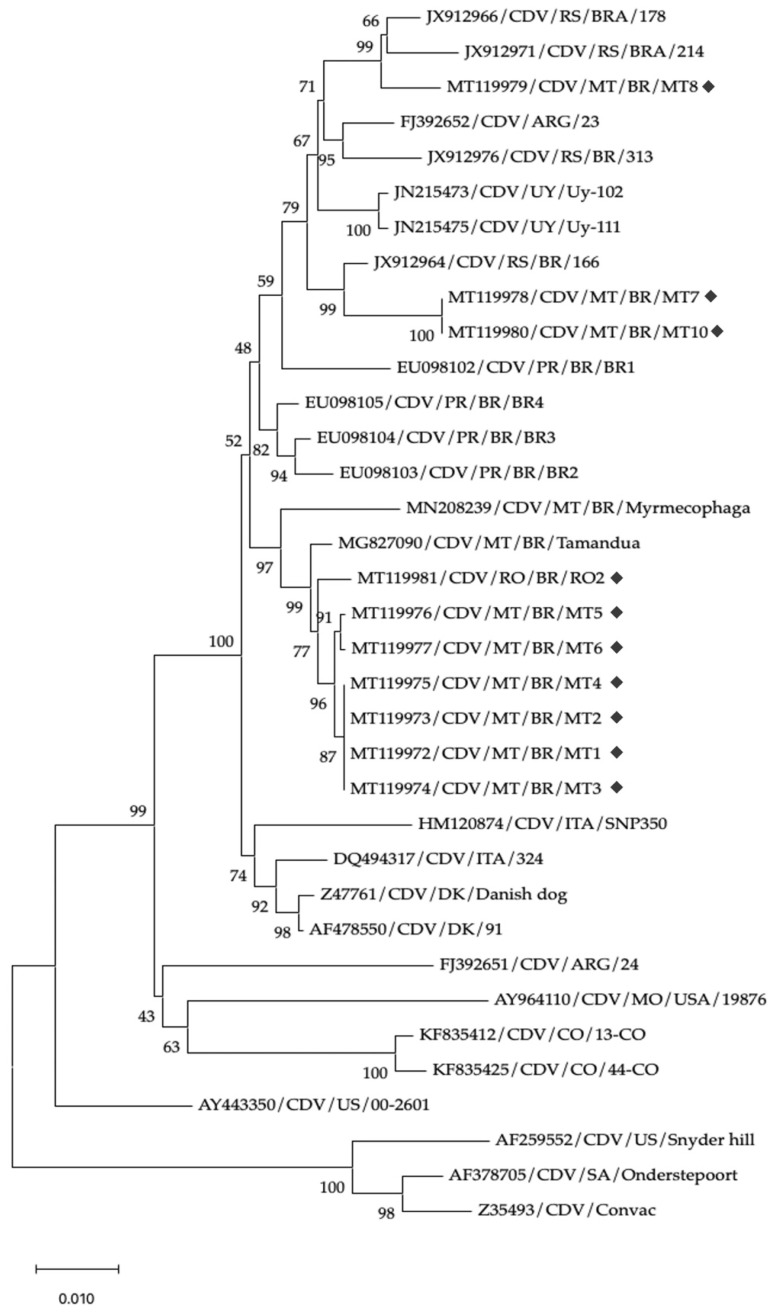
Phylogenetic tree constructed using the Neighbor-Joining (NJ) method based on 35 nucleotide sequences of the *H* gene, including 10 sequences generated in this study. Taxa are labeled with GenBank accession number, virus designation (*CDV*), geographic origin (state or country), and isolate name. Geometric shapes were used to highlight the CDV isolates generated in this study. The analysis was performed in MEGA X using 1696 aligned positions. Evolutionary distances were estimated using the Kimura 2-parameter model and are expressed as the number of base substitutions per site. The codon positions included were the 1st, 2nd, 3rd, and non-coding sites. Ambiguous positions were removed using pairwise deletion. Clade support values are based on 1000 bootstrap replicates.

A phylogenetic analysis indicated that our sequences clustered into two clades within the South America I/Europe lineage, supported by the maximum bootstrap value (100) (Figure 1). In the first clade, the MT8 sequence clustered with isolates from Southern Brazil, Argentina, and Uruguay. Strains MT7 and MT10 formed a subgroup closely related to a sequence previously detected in Rio Grande do Sul state. In the second clade, samples MT1, MT2, MT3, MT4, MT5, MT6, MT7, MT10, and RO2 diverged from the first group, with a minimum bootstrap value of 52, but were consistently grouped in a well-supported clade (bootstrap value of 97). This clade comprised strains detected in the present study as well as isolates obtained from animals of the order *Pilosa* (isolates BR/Mymercophaga and BR/Tamandua).

**Figure 2:**
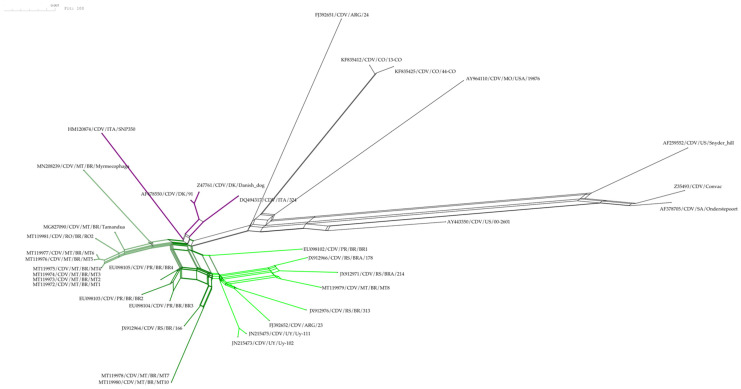
Reticulate phylogenetic network of canine distemper virus (CDV) isolates based on 35 H-gene sequences (1696 bp), including 10 obtained in this study. The network was constructed using the Neighbor-Net method in SplitsTree CE v6.3.30 with the P-distance model and 100 bootstrap replicates. Each node represents a unique CDV isolate, labeled with its GenBank accession number, geographic origin, and isolate name. Colors represent lineage affiliations: green and purple shades indicate clusters within the South America I/Europe lineage, while black indicates other lineages. Reticulations reflect phylogenetic conflicts possibly due to recombination or homoplasy.

**Figure 3:**
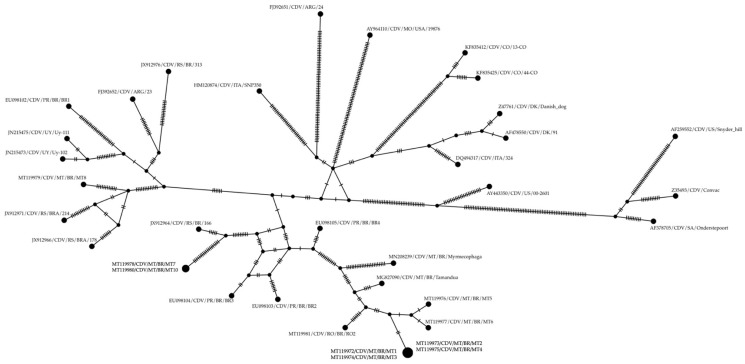
Median-joining haplotype network of 35 CDV H-gene sequences (1696 bp) constructed using PopART v1.7 with ε = 0. Each node represents a unique haplotype, and node size is proportional to the number of sequences sharing that haplotype. Black dots (median vectors) represent hypothetical or unsampled haplotypes. Branch lengths reflect the number of mutational steps between haplotypes. Brazilian strains are represented throughout the network, with special emphasis on the ten sequences generated in this study: MT1–MT8 and MT10 (from Mato Grosso) and RO1 (from Rondônia), which are distributed across distinct haplotype clusters, illustrating the genetic diversity and regional distribution of circulating CDV strains.

The haplotype networks (Figure 2 and Figure 3) show consistent groupings that are congruent with those observed in the phylogenetic tree (Figure 1), especially in the following clades: the MT/RO/Pilosa group, which displays the amino acid pattern 530S, 542N, and 549Y; the Southern Cone group (UY/ARG/RS/PR), characterized by the pattern 530G, 542I, and 549Y—except for strain RS/BRA/178, which presented residue 549H. Strain MT8 clustered within this latter group and appears to play an intermediary role among Uruguayan, Argentine, and other Brazilian strains, as it occupies a central position in the network of interactions between these isolates (Figure 2). On the other hand, strains MT7 and MT10, also belonging to this group, stand out for forming parallel interactions and networks in proximity to isolates RS/BR/166, PR/BR/BR2, BR3, and BR4.

The haplotypes of the European, North America II and III, South America II and III lineages, as well as the vaccine strains, exhibited divergent positions compared with the genotypes of the South America I/Europe lineage. These were characterized by numerous branches and long genetic distances, occupying positions opposite to the groups corresponding to the strains analyzed in the present study.

The majority of CDV isolates from dogs (7 out of 10; MT1, MT2, MT3, MT4, MT5, MT6, and RO2) presented serine (S), asparagine (N), and tyrosine (Y) at positions 530, 542, and 549, respectively. Except for the isolate from Rondônia state (RO), all other isolates were detected in the metropolitan area of Cuiabá and the Pantanal regions of Mato Grosso state (Poconé). Positions 530, 542, and 549 in the amino acid sequences of the isolates detected in anteaters (*Tamandua tetradactyla* and *Myrmecophaga tridactyla*) were identical to those found in most of the CDV isolates from dogs in Cuiabá and the Pantanal (530S/542N/549Y). Three CDV isolates from Sinop, located in the northern region of Mato Grosso (MT7, MT8, and MT10), exhibited a combination of glycine (G), isoleucine (I), and tyrosine (Y) at the same positions. Arginine (R) was conserved at position 519 in all sequences analyzed, except for the North American strain AY443350/CDV/USA/00-2601, which exhibited a substitution to isoleucine (I) at this site. Supplementary Figure S1 summarizes the amino acid residues identified at positions 530, 542, and 549 of the H gene in the analyzed CDV isolates.

## 4. Discussion

This study evaluated samples from dogs naturally infected with CDV in the states of Mato Grosso (nine samples) and Rondônia (one sample). Although CDV had been previously detected in the state of Mato Grosso (MT) [14], this is the first report describing the nucleotide sequences of the N and H genes in naturally infected dogs from this state, which is located precisely in the center of South America. Our study also provides the first report of CDV genotyping in the state of Rondônia. A previous investigation failed to detect positive samples from this region, despite identifying several cases in other areas of Brazil [14]. Notably, our study prioritized brain tissue from dogs with neurological symptoms, which likely increased the probability of detecting positive cases; therefore, the isolates are considered potentially neurotropic. Although the aim of the present study was not to characterize the specific cell types involved in the infections, previous studies indicate that CDV can infect multiple CNS cytotypes, with affinity for oligodendrocytes and neurons; oligodendrocytes predominate in subacute phases and are associated with demyelination, while neuronal infection may contribute to persistence and chronic signs [2,32]. Astrocytes and microglial cells may also be affected, albeit less frequently [33], supporting a multicellular tropism that adds complexity to CDV neuropathogenesis.

Initially, to confirm CDV infection, a protocol was used to partially amplify the nucleocapsid (N) gene, as it represents a conserved genetic fragment of the virus. The nucleocapsid gene sequences evaluated in this study confirmed the expected conserved pattern, consistent with the low variability typically observed in internal proteins of CDV [18].

The positive samples were subsequently reassessed through amplification of the hemagglutinin (H) gene, the genetic variability of which allows for the distinction of CDV lineages. Among the samples previously confirmed via N-gene amplification, ten were successfully amplified for the H gene and sequenced. The failure to amplify the remaining samples (*n* = 4) may be attributed either to genetic variability preventing primer binding or to RNA degradation that impaired amplification. This limitation is particularly relevant given that the H gene was amplified in multiple overlapping fragments ranging from 253 to 870 base pairs, which must be aligned to reconstruct the complete sequence. Furthermore, the presence of native or mutant strains lacking complementarity with the primers used in this study cannot be ruled out. Future studies employing updated primer sets or next-generation sequencing could help overcome these limitations and recover complete H-gene sequences from genetically divergent strains.

Our H-gene analysis places all sequences within the South America I/Europe lineage; however, beyond corroborating prior reports for South America [14,16], it adds novel evidence from Central–Western Brazil: the MT/RO isolates resolve into two well-supported clusters: one grouping with strains from Uruguay/Argentina and another with southern Brazilian strains. Importantly, we documented the circulation of distinct H-protein residue profiles at positions 530/542/549 (predominantly 530S/549Y, with 530G/549Y also present); in the literature, these patterns are linked to receptor usage and host range. These results expand the geographic coverage of CDV molecular data in Brazil and demonstrate co-circulation of divergent SA-I/Europe groups in MT/RO, refining the regional transmission picture and informing surveillance and vaccine discussions.

As illustrated by the haplotype network analyses, the MT8 isolate, for example, clustered among Uruguayan, Argentine, and Brazilian strains, and appears to play an intermediary role, occupying a central position within the network (Figure 2). Notably, this interaction is supported by high bootstrap values (Figure 1; Supplementary Figure S2). MT7, MT8, and MT10 were obtained from dogs in Sinop (Northern Mato Grosso). In the H-gene phylogeny, these sequences cluster with South America I/Europe strains from Southern Brazil (Paraná, Rio Grande do Sul) and Uruguay/Argentina, indicating close genetic relatedness. The sampled municipalities are part of the recent colonization front of Northern Mato Grosso, which were largely established by migrants from Southern Brazil in the 1970s–1980s along the BR-163 corridor (e.g., Sinop founded in 1974), providing historical context compatible with south-to-north introductions [34]. Nevertheless, the putative movement route cannot be confirmed from our data alone and should be validated with additional genomic and epidemiological evidence.

On the opposite side of the Europe/South America I clade, the remaining isolates formed a parallel branch composed of sequences generated in this study, along with isolates detected in mammals of the order *Pilosa*. Although one of these isolates originated from the state of Rondônia, the others were all collected in the metropolitan region of the Cuiabá River, encompassing the cities of Cuiabá and Poconé. Following the same reasoning regarding migratory flows between northern Mato Grosso and Southern Brazil, the southern portion of Rondônia is geographically and socioeconomically connected to the South–Western region of Mato Grosso—precisely where the municipalities of Poconé and Cuiabá are located. Based on our results, we propose the existence of two possible migratory flows that may have contributed to the dissemination of distinct CDV subgenotypes, as previously suggested by Budaszewski et al. [14] and Duque-Valencia et al. [16].

These two groups within the European/South American clade differ in the amino acid positions located at residues 530, 542, and 549. The sequences of isolates MT7, MT8, and MT10 present glycine (G), isoleucine (I), and tyrosine (Y), and stand out due to intense interactions and mutations, as observed in the reticulate and haplotype networks (Figure 2 and Figure 3). As seen in the networks, this group is positioned closer to the Europe and South America II and III lineages, whereas the other sequences are positioned more distantly, to the left of the networks, characterized by the amino acids serine (S), asparagine (N), and tyrosine (Y) at residues 530, 542, and 549. This last subgenotype predominated among dogs from the South–Western region and appears to have the potential to infect various wild mammal species, including those of the order Pilosa [4,5]. This finding substantiates several serological findings of CDV in local wild species such as *Cerdocyon thous*, *Chrysocyon brachyurus*, *Procyon cancrivorus*, *Puma concolor*, and *Leopardus pardalis* [35,36].

In particular, substitutions at sites 530 and 549 predominantly occur in CDV isolates obtained from novel host species, indicating that the spread of CDV to non-dog hosts is associated with evolution at these sites. However, at least in the Cuiabá region, the isolate infecting dogs is also infecting anteaters [4,5,37,38]. Given that all sequences belong to the South America I/Europe lineage and harbor substitutions at H-protein residues 530 and 549, these changes may contribute to reduced effectiveness of America I-based vaccines, potentially leading to vaccine failures [13], which may contribute to the high CDV infection rates in the study region as well as spillover to wildlife. Although we did not evaluate the vaccine efficacy, the divergence observed at residue 530—distinct from those found in vaccine strains—may have implications for immune recognition and warrants further investigation into potential impacts on vaccine protection [37,38].

## 5. Conclusions

The results of this study demonstrate that two genetic groups of the South America I/Europe lineage co-circulate in dogs from Mato Grosso and Rondônia and cluster with strains from Southern Brazil, Uruguay, and Argentina, indicating regional connectivity within a broader South American transmission network. The H-gene profiles identified—particularly residue combinations at 530/542/549 (predominantly 530S/549Y, with 530G/549Y also detected)—have been linked in the literature to receptor usage and host range, highlighting the need to evaluate vaccine representativeness and strengthen molecular surveillance at the dog–wildlife interface. These findings are consistent with our hypothesis of co-circulating SA-I/Europe groups and provide a framework for broader, cross-border monitoring and comparative genomics in South America. Inference about wildlife infection risk and vaccine performance remains provisional and should be confirmed via targeted pathogenesis and effectiveness studies. Importantly, our results underscore the need for continuous surveillance at wildlife–domestic animal interfaces, especially in ecologically sensitive and biodiverse regions such as Central–Western Brazil.

## Figures and Tables

**Table 1:** Primer sequences used for the amplification and sequencing of the N and H genes of canine distemper virus (CDV) from samples collected in the states of Mato Grosso (MT) and Rondônia (RO), Brazil.

Primer	Primer Nucleotide Sequence (5′–3′)	Gene	Base Pair	Reference
CDV-1F	ACAGGATTGCTGAGGACCTAT	N	287	Frisk et al. [18]
CDV-2R	CAAGATAACCATGTACGGTGC	N		Frisk et al. [18]
RH3-F *	AGGGCTCAGGTACTCCAGC	H	1937	Harder et al. [19]
RH4-R *	AATGCTAGAGATGGTTTAATT	H		Harder et al. [19]
H1F **	ATGCTCTCCTACCAAGACAA	H	789	An et al. [17]
H1R **	CATGTCATTCAGCCACCGTT	H		An et al. [17]
H2F **	AATATGCTAACCGCTATCTC	H	523	An et al. [17]
H2RB **	TTTGGTTGCACATAGGGTAG	H		Budaszewski et al. [14]
H3FB **	CATATGATATATCCCGGGGC	H	253	Budaszewski et al. [14]
H3R **	TCARGGWTTTKAACGRYYAC	H		An et al. [17]
CDVF10B **	TAYCATGAYAGYARTGGTTC	H	870	Hashimoto et al. [20]
CDVR10 **	ARTYYTCRACACTGRTKGTG	H		Hashimoto et al. [20]

* Primers used in the first-round RT-PCR; ** primers used in the nested PCR for H-gene amplification.

## Data Availability

Data available in a publicly accessible repository. The data presented in this study are openly available in the GenBank database under accession numbers MT119972–MT119992. [GenBank] [https://www.ncbi.nlm.nih.gov/genbank/] [MT119972–MT119992] (accessed on 20 July 2025).

## Supplementary Materials

The following supporting information can be downloaded from https://www.mdpi.com/article/10.3390/vetsci12100948/s1. Figure S1: Amino acid residues at positions 530, 542, and 549 of the H protein of CDV isolates. The figure displays a heatmap summarizing the identity and distribution of residues at the evaluated positions. Each row corresponds to a CDV isolate, identified by its GenBank accession (or sequence ID), and columns represent the respective amino acid positions. Residues are color-coded according to the legend shown below the heatmap. Figure S2: Reticulate phylogenetic network of 35 CDV isolates based on the full-length H gene (1696 bp), constructed using the Neighbor-Net method in SplitsTree CE v6.3.30. Pairwise genetic distances were calculated using the P-distance method, and network robustness was evaluated using bootstrap analysis with 100 replicates; only values ≥70 are displayed on the major branches. Bootstrap values are shown along major branches, supporting key phylogenetic splits among isolates. The network topology reflects conflicting phylogenetic signals and possible recombination events. The CDV strains sequenced in this study (MT1–MT8, MT10, and RO1) cluster within the South America/Europe I lineage alongside previously described strains from Brazil, Uruguay, and Argentina. Table S1: CDV reference sequences used in phylogenetic analyses.
