# Supplementary material for: Characterization of the Hemagglutinin Gene of Morbillivirus canis in Domestic Dogs from the Mid-Western Area of Brazil
Source: Vet Sci. 2025 Sep 30;12(10):948. doi: 10.3390/vetsci12100948 (PMC12567662; doi:10.3390/vetsci12100948)
Supplement: Supplementary file 1 [file vetsci-12-00948-s001.zip › vetsci-3894044-supplementary.pdf]

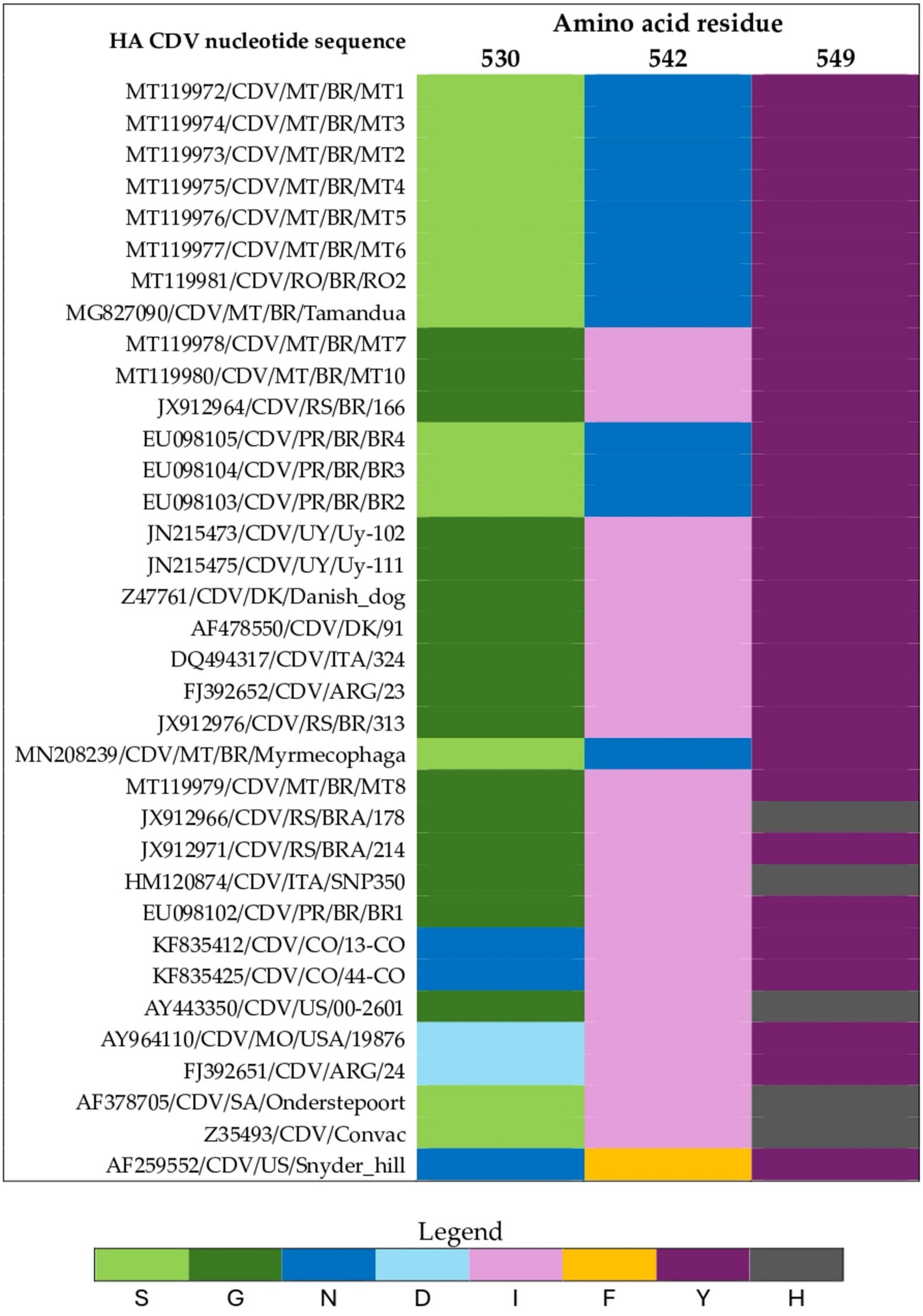

Figure S1: Amino acid residues at positions 530, 542, and 549 of the H protein of CDV isolates. The figure displays a heatmap summarizing the identity and distribution of residues at the evaluated positions. Each row corresponds to a CDV isolate, identified by its GenBank accession (or sequence ID), and columns represent the respective amino acid positions. Residues are color-coded according to the legend shown below the heatmap.

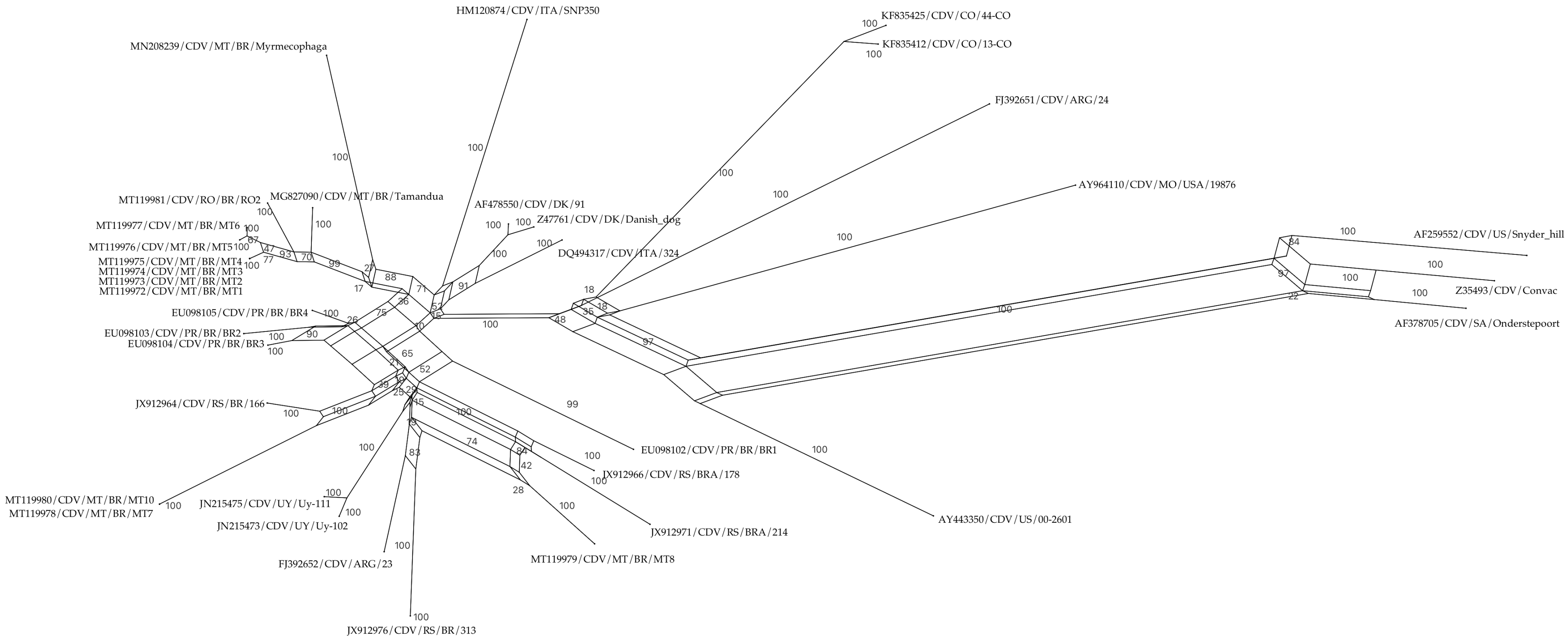

Figure S2: Reticulate phylogenetic network of 35 CDV isolates based on the full-length H gene (1696 bp), constructed using the Neighbor-Net method in SplitsTree CE v6.3.30. Pairwise genetic distances were calculated using the P-distance method, and network robustness was evaluated using bootstrap analysis with 100 replicates; only values ≥70 are displayed on the major branches. Bootstrap values are shown along major branches, supporting key phylogenetic splits among isolates. The network topology reflects conflicting phylogenetic signals and possible recombination events. The CDV strains sequenced in this study (MT1–MT8, MT10, and RO1) cluster within the South America/Europe I lineage alongside previously described strains from Brazil, Uruguay, and Argentina.

Table S1: CDV reference sequences used in phylogenetic analyses.

GenBank Accession	Location	Host	Isolate ID
MT119972	Mato Grosso State, Brazil	Domestic dog	MT1
MT119973	Mato Grosso State, Brazil	Domestic dog	MT2
MT119974	Mato Grosso State, Brazil	Domestic dog	MT3
MT119975	Mato Grosso State, Brazil	Domestic dog	MT4
MT119976	Mato Grosso State, Brazil	Domestic dog	MT5
MT119977	Mato Grosso State, Brazil	Domestic dog	MT6
MT119978	Mato Grosso State, Brazil	Domestic dog	MT7
MT119979	Mato Grosso State, Brazil	Domestic dog	MT8
MT119980	Mato Grosso State, Brazil	Domestic dog	MT10
MT119981	Rondônia State, Brazil	Domestic dog	RO2
MG827090	Mato Grosso State, Brazil	Collared anteater	Tamandua
MN208239	Mato Grosso State, Brazil	Giant anteater	Myrmecophaga
FJ392652	Argentina	Domestic dog	ARG/23
JN215473	Uruguay	Domestic dog	UY/102
FJ392651	Argentina	Domestic dog	ARG/24
JN215475	Uruguay	Domestic dog	UY/111
JX912966	Rio Grande do Sul State, Brazil	Domestic dog	RS/BRA/178
JX912964	Rio Grande do Sul State, Brazil	Domestic dog	RS/BRA/166
EU098103	Paraná State, Brazil	Domestic dog	PR/BRA/BR2
EU098104	Paraná State, Brazil	Domestic dog	PR/BRA/BR3
EU098105	Paraná State, Brazil	Domestic dog	PR/BRA/BR4
HM120874	Italy	Red fox	SNP350
DQ494317	Italy	Domestic dog	324
KF835412	Colombia	Domestic dog	13-CO
KF835425	Colombia	Domestic dog	44-CO
AY443350	United States of America	Raccoon	2601
AY964110	United States of America	Domestic dog	19876
Z47761	Denmark	Domestic dog	Danish dog
AF478550	Denmark	Infected Vero cells	91
AF259552	United States of America	Domestic dog/Vaccine	Snider Hill
AF378705	South Africa	Domestic dog/Vaccine	Onderstepoort
Z35493	Asia (South Korea)	Domestic dog/Vaccine	Convac
